# Molecular proof for *Lophomonas* infection in a patient with history of breast cancer

**DOI:** 10.1002/rcr2.1027

**Published:** 2022-08-27

**Authors:** Ahmad Shafahi, Ali Sharifpour, Erfan Ghadirzadeh, Amirmasoud Taheri, Mahdi Fakhar, Mostafa Soleymani

**Affiliations:** ^1^ Department of Internal Medicine, Cardiovascular Research Center, Institute of Basic and Clinical Physiology Sciences Kerman University of Medical Sciences Kerman Iran; ^2^ Pulmonary and Critical Care Division, Imam Khomeini Hospital, Iranian National Registry Center for Lophomoniasis (INRCL) Mazandaran University of Medical Sciences Sari Iran; ^3^ Iranian National Registry Center for Lophomoniasis (INRCL), Imam Khomeini Hospital Mazandaran University of Medical Sciences Sari Iran

**Keywords:** breast cancer, *Lophomonas*, Lophomoniasis, PCR

## Abstract

We report a 52‐year‐old patient with a history of breast cancer (BC) referred to the clinic of Afzalipour Hospital, in Kerman, eastern Iran, with a 1‐week complaint of restless dyspnea. A chest computed tomography scan revealed consolidations in the upper lobes of both lungs. The patient had no history of smoking or underlying diseases that would predispose her to consolidation, including pneumonia. Then, bronchoscopy was performed for the patient, and bronchoalveolar lavage fluid was sent to the Iranian National Registry Center, where the *Lophomonas* parasite was detected in the sample and confirmed using a polymerase chain reaction test. Finally, her symptoms improved by receiving oral metronidazole three times a day for 3 weeks.

## Graphical abstract

We report a patient with a history of breast cancer with a 1‐week complaint of at rest dyspnea. Chest computed tomography scan revealed consolidations in the upper lobe of both lungs. Bronchoscopy was performed for the patient and Lophomonas parasite was detected. Her symptoms improved by receiving oral metronidazole three times a day for 3 weeks.

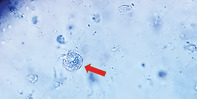

## INTRODUCTION

Breast cancer (BC) is the second leading cause of cancer death in women in the United States, accounting for nearly 40,000 deaths each year, and is the most commonly diagnosed cancer in females globally.^1^ The immune system is suppressed in the early stages of this cancer, both during the disease by alterations in T‐cells and as a result of chemotherapy drugs, such as docetaxel and trastuzumab.^2^, ^3^, ^4^ Febrile neutropenia and many opportunistic infections can develop during this immunosuppression, including candidiasis and parasitic infections such as *Toxoplasma gondi*.^4^, ^5^, ^6^ *Lophomonas blattarum* (*L*. *blattarum*) is an anaerobic protozoan cell which inhabits cockroaches' guts. Its cysts can be found in the faeces of these insects, and humans are usually infected through inhalation of cyst‐containing aerosols. *L*. *blattarum* is the main cause of lophomoniasis, a parasitic chronic respiratory infection which results in fever, pneumonia and chronic expectorating coughs.^7^, ^8^ This parasite has been observed to infect those with a suppressed immune system^9^ and to cause lung cavities.^10^

Considering its effects on the respiratory system, particularly in immunocompromised patients, and the fact that it can simply be treated with metronidazole, it is essential to be aware of this protozoa and treat it effectively. Herein, we report a case of *Lophomonas* infection in a BC patient.

## CASE REPORT

On 2021, a 52‐year‐old housewife with underlying BC was referred to the Afzalipour Hospital, Kerman, Kerman province, eastern Iran, with a 1‐week complaint of dyspnea at rest. She had not yet taken any medications. She had no history of asthma or cardiovascular disease such as myocardial infarction, did not smoke or abuse drugs, and had no history of asbestos exposure, trauma, weight loss, or night sweats. She was conscious and oriented. Vital signs included (BP: 130/90 mmHg, HR: 98 beats/min, RR: 20, *T*: 38.8°C, SpO_2_: 94%). She had crackles in both lungs during the physical examination, while other organ examinations were unremarkable.

The patient's primary laboratory tests, which included CBC, fasting blood sugar, Na, K, Cr, TSH, LFT, ECG and other laboratory tests, were normal except a slight increase in levels of ESR, CRP and a mild decrease in haemoglobin (Table 1). A COVID‐19 real time polymerase chain reaction (RT‐PCR) test was requested, but the results were negative. Blood and sputum cultures were negative. When a chest radiograph revealed suspicious consolidations, a chest computed tomography (CT) scan was ordered, which revealed consolidations in the upper lobes of both lungs. For further evaluation, we requested a pulmonologist consultation. The pulmonologist advised performing a bronchoscopy because of the underlying BC.

**TABLE 1:** Patient's laboratory data results

Lab data parameter	Result	Normal range
pH^a^	7.41	7.35–7.45
pCO_2_ ^a^	53 mmHg	35–45
HCO_3_ ^a^	31 mmol/L	24–32
Triglyceride	96 mg/dl	<200
Cholesterol	115 mg/dl	<200
TSH	2.8 μIU/ml	0.4–5.5
FBS	87 mg/dl	70–110
AST	24 U/L	5–40
ALT	20 U/L	<45
ALP	208 U/L	80–306
WBC	9400 U/μl	4400–11,000
RBC	4.2 × 10^6^ /μl	4.2–5.4
Haemoglobin	10.9 g/dl	12–15
Platelet	740,000	145,000–450,000
Haematocrit	35.2	35.5–44.9
Neutrophils	79%	55–70
Lymphocytes	17%	20–35
Monocytes	3%	3–8
ESR	45 mg/h	0–20
CRP	40 mg/L	<6
Urea	29 mg/dl	13–43
Creatinine	0.8 mg/dl	0.6–1.3
Na	139 mEq/L	135–145
K	4.6 mEq/L	3.5–5.5
Mg	2.1 mg/dl	1.8–2.5

Abbreviations: TSH, thyroid stimulating hormone; FBS, fasting blood sugar; AST, aspartate transaminase; ALP, alkaline phosphatase; WBC, white blood cell; RBC, red blood cell; ESR, erythrocyte sedimentation rate; CRP, C‐reactive protein.

^a^ pH, pCO_2_ and HCO_3_ results were obtained from venous blood gas (VBG), not arterial blood gas (ABG).

**FIGURE 1:**
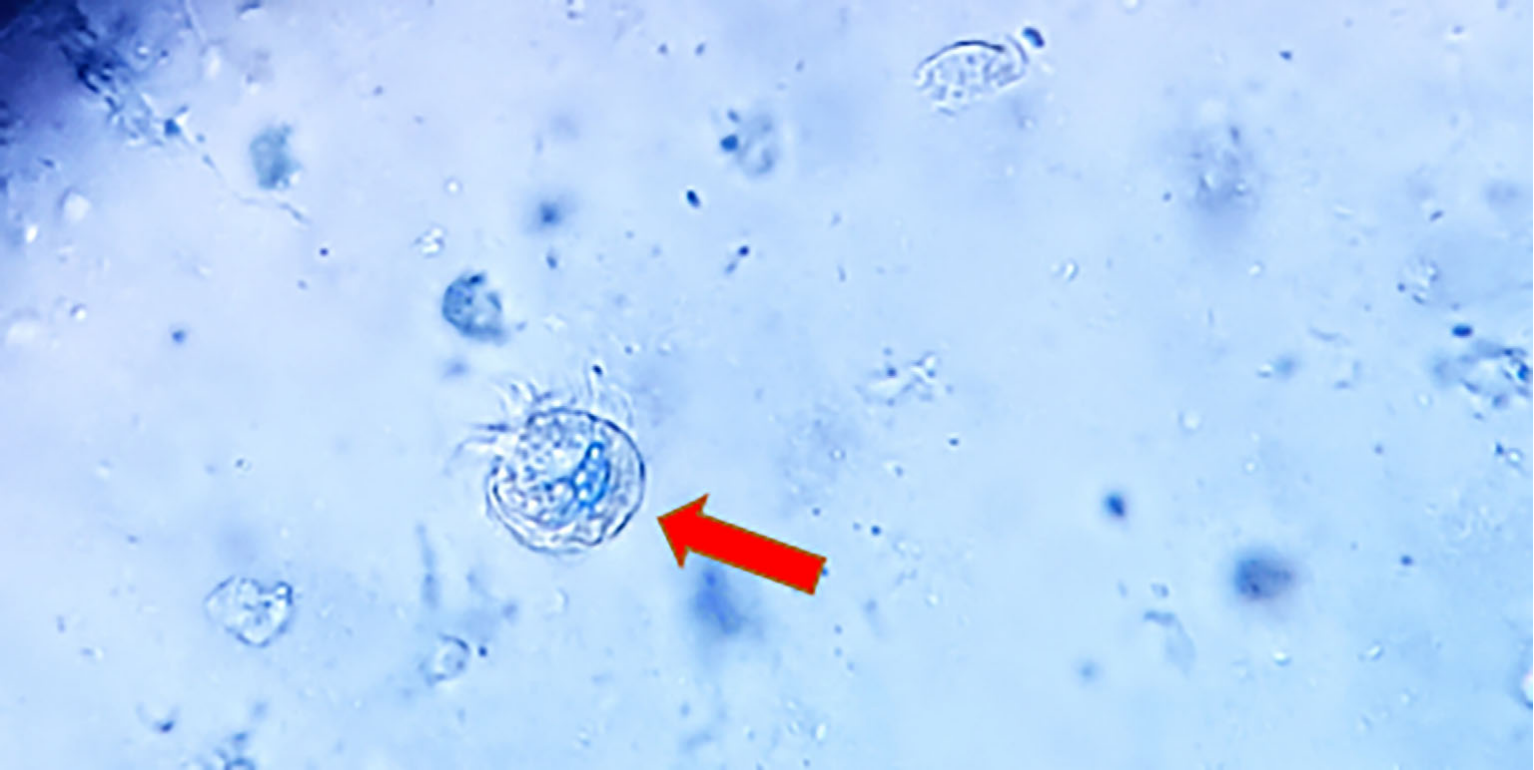
Wet mount smear micrograph of BAL specimen showing *Lophomonas* trophozoite (a head of arrow) with polar tuft of flagella (magnification ×400).

A bronchoscopy was performed on the patient, and bronchoalveolar lavage fluid (BALF) samples were collected. One sample was sent to the microbiology laboratory to check for bacterial infection, and another sample was sent to Iranian National Registry Center for Lophomoniasis (INRCL) to examine for *Lophomonas* parasite. A wet smear prepared from BALF sediment revealed the presence of a few *Lophomonas* live and motile flagellated parasites (Figure 1). In the meantime, a conventional PCR test^8^ was performed on the sample, which confirmed the microscopic results. This patient received oral metronidazole 500 mg every 8 h for 3 weeks, which improved the patient's symptoms. On a 3‐month follow‐up, the patient had no respiratory complaints and her physical examination and laboratory tests were normal.

## DISCUSSION

Consolidations in the lungs are caused by different diseases, such as infections, infarction and contusion, malignancies such as lymphomas, haemorrhages, and rheumatologic and immunologic disorders.^11^, ^12^, ^13^ Most commonly, infectious diseases, such as pneumonia are the source, and among them, bacteria, fungi and protozoa, are the most common pathogens.^14^, ^15^ This patient had risk factors for *Lophomonas* infection, including immunosuppressed status and contact with cockroaches in her house.^8^, ^16^

Our report is consistent with other reports which suggest that *Lophomonas* is more common in immunosuppressed patients. A study by He et al.^17^ reported two cases of lophomoniasis in transplant recipients. Also, Woerden et al.^18^ found that flagellated protozoa in sputum are more prevalent in asthmatic patients who received corticosteroids compared to the control group. Wahid et al.^19^ presented *Lophomonas* in a 29‐year‐old female with systemic lupus erythromatosus. Although many studies have reported eosinophilia as the main result in CBC, but it was not seen in our patient which could be due to her immunocompromised state,^16^, ^20^ which is consistent with Wahid et al. report.^19^ *Lophomonas* can be diagnosed under light microscopy, but it is hard for an unskilled parasitologist to differentiate between *Lophomonas* and normal bronchial epithelium due to their analogy.^8^, ^16^, ^21^ But our colleagues have demonstrated a molecular PCR based diagnosis with more sensitivity and more specificity.^8^, ^22^ Our patient's symptoms improved with metronidazole administration. This drug is also effective in treating anaerobic respiratory infections and those infections could have been a probable cause, but her laboratory tests and cultures for bacterial infections, both aerobic and anaerobic, were negative.

This case presentation strongly persuade us to pay additional deliberation to this newly emerging pulmonary pathogen. As a conclusion, lophomoniasis should be considered in the differential diagnosis of each patient with lung consolidations, especially in immunocompromised conditions such as malignancies. Therefore, extra attention should be focused on pulmonary consolidations in patients with BC.

## AUTHOR CONTRIBUTION

Mahdi Fakhar and Ahmad Shafahi involved in interpretation and collecting of data. Ali Sharifpour, Amirmasoud Taheri and Erfan Ghadirzadeh were writing and editing of the manuscript. Mahdi Fakhar and Mostafa Soleymani involved in editing and preparing the final version of manuscript. All authors reviewed the paper and approved the final version of the manuscript.

## CONFLICT OF INTEREST

None declared.

## ETHICS STATEMENT

The authors declare that appropriate written informed consent was obtained for the publication of this manuscript and accompanying images.

## Data Availability

The data that support the findings of this study are available on request from the corresponding author.

## References

[1] Siegel RL , Miller KD , Jemal A . Cancer statistics, 2016. CA Cancer J Clin. 2016;66(1):7–30. 26742998 10.3322/caac.21332

[2] de Oliveira CE , Santiago KB , Conti B , Conte FL , Tasca KI , Romagnoli GG , et al. Brazilian green propolis: a novel tool to improve the cytotoxic and immunomodulatory action of docetaxel on MCF‐7 breast cancer cells and on women monocyte. Phytother Res. 2022;36(1):448–61. 34862831 10.1002/ptr.7345

[3] Rye IH , Huse K , Josefsson SE , Kildal W , Danielsen HE , Schlichting E , et al. Breast cancer metastasis: immune profiling of lymph nodes reveals exhaustion of effector T cells and immunosuppression. Mol Oncol. 2022;16(1):88–103. 34165864 10.1002/1878-0261.13047 PMC8732351

[4] Funakoshi T , Suzuki M , Muss HB . Infection risk in breast cancer patients treated with trastuzumab: a systematic review and meta‐analysis. Breast Cancer Res Treat. 2015;149(2):321–30. 25385179 10.1007/s10549-014-3184-3

[5] Teoh F , Pavelka N . How chemotherapy increases the risk of systemic candidiasis in cancer patients: current paradigm and future directions. Pathogens. 2016;5(1):6. 10.3390/pathogens5010006 PMC4810127 26784236

[6] Kalantari N , Darabi ZA , Siadati S , Nikbakhsh N , Ghasemi M , Ghaffari T , et al. Detection of *Toxoplasma gondii* DNA in malignant breast tissues in breast cancer patients. Int J Mol Cell Med. 2017;6(3):190–6. 29682491 10.22088/acadpub.BUMS.6.3.190 PMC5898643

[7] Zhang X , Xu L , Wang LL , Liu S , Li J , Wang X . Bronchopulmonary infection with *Lophomonas blattarum*: a case report and literature review. J Int Med Res. 2011;39(3):944–9. 21819728 10.1177/147323001103900329

[8] Fakhar M , Sharifpour A , Nakhaei M , Banimostafavi ES , Ghasemi M , Abedian S , et al. *Lophomonas* and lophomoniasis. Gorgan, Iran: Nourozi Publisher; 2021.

[9] Sharifpour A , Zarrinfar H , Fakhar M , Zakariaei Z , Soleymani M , Banimostafavi ES , et al. First report of Lophomonas infection in a patient with AML‐2 from Qeshm Island, Persian gulf, southern Iran. Respirol Case Rep. 2022;10(2):e0906. 35127100 10.1002/rcr2.906 PMC8792117

[10] Taheri A , Fakhar M , Sharifpour A , Banimostafavi ES . Cavitary pulmonary lesions following emerging lophomoniasis: a novel perspective. Respirol Case Rep. 2022;10(3):e0908. 35140977 10.1002/rcr2.908 PMC8812051

[11] De Maddi F , Cinelli R , Rigante D , Mazzarella G , Siani P . Lung parenchymal consolidation as an uncommon presentation and cause of delayed diagnosis in atypical Kawasaki syndrome. Rheumatol Int. 2009;29(11):1373–6. 19116718 10.1007/s00296-008-0830-2

[12] Rockall AG , Hatrick A , Armstrong P , Wastie M . Diagnostic imaging. 7th ed, Sussex, UK, John Wiley & Sons Publisher; 2013.

[13] Wong A , Koutsogiannis Z , Greene SL . Pulmonary haemorrhage from therapeutic rivaroxaban use: chest radiograph consolidation is not always infection! Emerg Med Australas. 2014;26(3):318–9. 24809972 10.1111/1742-6723.12235

[14] Afsar S , Choudhri AN , Ali J , Muneer AB . Primary pulmonary amoebiasis‐an unusual cause of pulmonary consolidation. J Pak Med Assoc. 1992;42:245. 1469769

[15] Kjeldsberg KM , Oh K , Murray KA , Cannon G . Radiographic approach to multifocal consolidation. Semin Ultrasound CT MRI. 2002;23(4):288–301. 10.1016/s0887-2171(02)90018-1 12465686

[16] Martinez‐Girón R , van Woerden HC . Lophomonas blattarum and bronchopulmonary disease. J Med Microbiol. 2013;62(11):1641–8. 23946475 10.1099/jmm.0.059311-0

[17] He Q , Chen X , Lin B , Qu L , Wu J , Chen J . Late onset pulmonary *Lophomonas blattarum* infection in renal transplantation: a report of two cases. Intern Med. 2011;50(9):1039–43. 21532229 10.2169/internalmedicine.50.4672

[18] Van Woerden HC , Ratier‐Cruz A , Aleshinloye OB , Martinez‐Giron R , Gregory C , Matthews IP . Association between protozoa in sputum and asthma: a case‐control study. Respir Med. 2011 Jun 1;105(6):877–84. 21138788 10.1016/j.rmed.2010.11.016

[19] Wahid W , Fahmi NA , Salleh AF , Yasin AM . Bronchopulmonary lophomoniasis: a rare cause of pneumonia in an immunosuppressed host. Respir Med Case Rep. 2019;28:100939. 31667075 10.1016/j.rmcr.2019.100939 PMC6812266

[20] Tyagi R , Anand KB , Teple K , Negi RS . Lophomonas blattarum infection in immunocompetent patient. Lung India: Off Organ Indian Chest Soc. 2016;33(6):667–8. 10.4103/0970-2113.192867 PMC5112827 27890999

[21] Willy D , Patricia C , Rainier O , Nestor L . Community‐acquired pneumonia caused by *Lophomona* sp. Community Acquired Infect. 2017;4(2):38.

[22] Fakhar M , Nakhaei M , Sharifpour A , Safanavaei S , Abedi S , Tabaripour R , et al. Morphological and molecular identification of emerged *Lophomonas blattarum* infection in Mazandaran Province, northern Iran: first registry‐based study. Acta Parasitol. 2021;66(4):1510–6. 34115281 10.1007/s11686-021-00422-3

